# Supplementary material for: Batch Size Effects on Mid‐2025 State‐of‐the‐Art Large Language Model Performance in Automated Title and Abstract Screening
Source: Cochrane Evid Synth Methods. 2026 Apr 11;4(3):e70082. doi: 10.1002/cesm.70082 (PMC13073229; doi:10.1002/cesm.70082)
Supplement: Supplementary file 1 — Supporting File 1: [file CESM-4-e70082-s001.docx]

**APPENDIX**

**Sensitivity**

| AI Model | Batch Size | Mean | Std Dev | Std Error | Lower 95% CI | Upper 95% CI |
| --- | --- | --- | --- | --- | --- | --- |
| Gemini-2.5-flash | 1 | 0.85 | 0.01 | 0.00 | 0.85 | 0.86 |
| Gemini-2.5-flash | 10 | 0.95 | 0.02 | 0.01 | 0.94 | 0.96 |
| Gemini-2.5-flash | 25 | 0.95 | 0.02 | 0.01 | 0.93 | 0.96 |
| Gemini-2.5-flash | 50 | 0.96 | 0.02 | 0.01 | 0.94 | 0.97 |
| Gemini-2.5-flash | 100 | 0.95 | 0.04 | 0.01 | 0.92 | 0.98 |
| Gemini-2.5-flash | 150 | 0.97 | 0.02 | 0.01 | 0.95 | 0.99 |
| Gemini-2.5-flash | 200 | 0.98 | 0.02 | 0.01 | 0.96 | 0.99 |
| Gemini-2.5-flash | 400 | 0.97 | 0.01 | 0.00 | 0.96 | 0.98 |
| Gemini-2.5-pro | 1 | 0.99 | 0.01 | 0.00 | 0.99 | 1.00 |
| Gemini-2.5-pro | 10 | 1.00 | 0.00 | 0.00 | 1.00 | 1.00 |
| Gemini-2.5-pro | 25 | 1.00 | 0.00 | 0.00 | 1.00 | 1.00 |
| Gemini-2.5-pro | 50 | 1.00 | 0.01 | 0.00 | 0.99 | 1.00 |
| Gemini-2.5-pro | 100 | 1.00 | 0.00 | 0.00 | 1.00 | 1.00 |
| Gemini-2.5-pro | 150 | 1.00 | 0.00 | 0.00 | 1.00 | 1.00 |
| Gemini-2.5-pro | 200 | 1.00 | 0.00 | 0.00 | 1.00 | 1.00 |
| Gemini-2.5-pro | 400 | 0.99 | 0.01 | 0.00 | 0.99 | 1.00 |
| Gemini-2.5-pro | 790 | 0.98 | 0.01 | 0.00 | 0.97 | 0.99 |
| GPT-5 mini (with thinking) | 1 | 0.98 | 0.01 | 0.00 | 0.97 | 0.98 |
| GPT-5 mini (with thinking) | 10 | 1.00 | 0.01 | 0.00 | 0.99 | 1.00 |
| GPT-5 mini (with thinking) | 25 | 1.00 | 0.01 | 0.00 | 0.99 | 1.00 |
| GPT-5 mini (with thinking) | 50 | 0.99 | 0.00 | 0.00 | 0.99 | 0.99 |
| GPT-5 mini (with thinking) | 100 | 0.99 | 0.00 | 0.00 | 0.99 | 0.99 |
| GPT-5 mini (with thinking) | 150 | 0.98 | 0.02 | 0.01 | 0.96 | 0.99 |
| GPT-5 mini (with thinking) | 200 | 0.88 | 0.10 | 0.03 | 0.81 | 0.95 |
| GPT-5 mini (with thinking) | 400 | 0.48 | 0.12 | 0.04 | 0.39 | 0.57 |
| GPT-5 (with thinking) | 1 | 0.95 | 0.02 | 0.01 | 0.94 | 0.96 |
| GPT-5 (with thinking) | 10 | 0.98 | 0.02 | 0.01 | 0.97 | 1.00 |
| GPT-5 (with thinking) | 25 | 0.98 | 0.01 | 0.00 | 0.97 | 0.99 |
| GPT-5 (with thinking) | 50 | 0.99 | 0.01 | 0.00 | 0.98 | 0.99 |
| GPT-5 (with thinking) | 100 | 0.97 | 0.01 | 0.00 | 0.96 | 0.98 |
| GPT-5 (with thinking) | 150 | 0.97 | 0.02 | 0.01 | 0.96 | 0.98 |
| GPT-5 (with thinking) | 200 | 0.98 | 0.01 | 0.00 | 0.98 | 0.99 |

**Specificity**

| AI Model | Batch Size | Mean | Std Dev | Std Error | Lower 95% CI | Upper 95% CI |
| --- | --- | --- | --- | --- | --- | --- |
| Gemini-2.5-flash | 1 | 0.97 | 0.00 | 0.00 | 0.97 | 0.97 |
| Gemini-2.5-flash | 10 | 0.94 | 0.01 | 0.00 | 0.94 | 0.95 |
| Gemini-2.5-flash | 25 | 0.94 | 0.00 | 0.00 | 0.94 | 0.94 |
| Gemini-2.5-flash | 50 | 0.94 | 0.00 | 0.00 | 0.93 | 0.94 |
| Gemini-2.5-flash | 100 | 0.93 | 0.01 | 0.00 | 0.92 | 0.94 |
| Gemini-2.5-flash | 150 | 0.93 | 0.02 | 0.01 | 0.92 | 0.95 |
| Gemini-2.5-flash | 200 | 0.93 | 0.01 | 0.00 | 0.92 | 0.94 |
| Gemini-2.5-flash | 400 | 0.94 | 0.01 | 0.00 | 0.93 | 0.95 |
| Gemini-2.5-pro | 1 | 0.97 | 0.00 | 0.00 | 0.97 | 0.97 |
| Gemini-2.5-pro | 10 | 0.95 | 0.00 | 0.00 | 0.95 | 0.95 |
| Gemini-2.5-pro | 25 | 0.95 | 0.00 | 0.00 | 0.95 | 0.96 |
| Gemini-2.5-pro | 50 | 0.95 | 0.01 | 0.00 | 0.94 | 0.95 |
| Gemini-2.5-pro | 100 | 0.95 | 0.01 | 0.00 | 0.95 | 0.96 |
| Gemini-2.5-pro | 150 | 0.96 | 0.01 | 0.00 | 0.95 | 0.96 |
| Gemini-2.5-pro | 200 | 0.95 | 0.01 | 0.00 | 0.94 | 0.95 |
| Gemini-2.5-pro | 400 | 0.93 | 0.02 | 0.00 | 0.92 | 0.94 |
| Gemini-2.5-pro | 790 | 0.91 | 0.02 | 0.01 | 0.90 | 0.93 |
| GPT-5 mini (with thinking) | 1 | 0.98 | 0.00 | 0.00 | 0.98 | 0.98 |
| GPT-5 mini (with thinking) | 10 | 0.97 | 0.01 | 0.00 | 0.97 | 0.98 |
| GPT-5 mini (with thinking) | 25 | 0.96 | 0.00 | 0.00 | 0.96 | 0.97 |
| GPT-5 mini (with thinking) | 50 | 0.96 | 0.01 | 0.00 | 0.96 | 0.96 |
| GPT-5 mini (with thinking) | 100 | 0.96 | 0.01 | 0.00 | 0.95 | 0.96 |
| GPT-5 mini (with thinking) | 150 | 0.96 | 0.01 | 0.00 | 0.96 | 0.97 |
| GPT-5 mini (with thinking) | 200 | 0.97 | 0.01 | 0.00 | 0.96 | 0.97 |
| GPT-5 mini (with thinking) | 400 | 0.92 | 0.15 | 0.05 | 0.81 | 1.00 |
| GPT-5 (with thinking) | 1 | 0.99 | 0.00 | 0.00 | 0.98 | 0.99 |
| GPT-5 (with thinking) | 10 | 0.98 | 0.00 | 0.00 | 0.98 | 0.99 |
| GPT-5 (with thinking) | 25 | 0.98 | 0.00 | 0.00 | 0.98 | 0.98 |
| GPT-5 (with thinking) | 50 | 0.98 | 0.00 | 0.00 | 0.98 | 0.98 |
| GPT-5 (with thinking) | 100 | 0.98 | 0.00 | 0.00 | 0.98 | 0.98 |
| GPT-5 (with thinking) | 150 | 0.98 | 0.00 | 0.00 | 0.98 | 0.98 |
| GPT-5 (with thinking) | 200 | 0.98 | 0.00 | 0.00 | 0.98 | 0.99 |

**Accuracy**

| AI Model | Batch Size | Mean | Std Dev | Std Error | Lower 95% CI | Upper 95% CI |
| --- | --- | --- | --- | --- | --- | --- |
| Gemini-2.5-flash | 1 | 0.96 | 0.00 | 0.00 | 0.95 | 0.96 |
| Gemini-2.5-flash | 10 | 0.94 | 0.01 | 0.00 | 0.94 | 0.95 |
| Gemini-2.5-flash | 25 | 0.94 | 0.01 | 0.00 | 0.94 | 0.94 |
| Gemini-2.5-flash | 50 | 0.94 | 0.00 | 0.00 | 0.94 | 0.94 |
| Gemini-2.5-flash | 100 | 0.93 | 0.01 | 0.00 | 0.92 | 0.94 |
| Gemini-2.5-flash | 150 | 0.94 | 0.02 | 0.01 | 0.93 | 0.95 |
| Gemini-2.5-flash | 200 | 0.93 | 0.01 | 0.00 | 0.93 | 0.94 |
| Gemini-2.5-flash | 400 | 0.94 | 0.01 | 0.00 | 0.93 | 0.95 |
| Gemini-2.5-pro | 1 | 0.97 | 0.00 | 0.00 | 0.97 | 0.97 |
| Gemini-2.5-pro | 10 | 0.96 | 0.00 | 0.00 | 0.95 | 0.96 |
| Gemini-2.5-pro | 25 | 0.96 | 0.00 | 0.00 | 0.96 | 0.96 |
| Gemini-2.5-pro | 50 | 0.95 | 0.01 | 0.00 | 0.95 | 0.96 |
| Gemini-2.5-pro | 100 | 0.96 | 0.01 | 0.00 | 0.95 | 0.96 |
| Gemini-2.5-pro | 150 | 0.96 | 0.01 | 0.00 | 0.96 | 0.97 |
| Gemini-2.5-pro | 200 | 0.95 | 0.01 | 0.00 | 0.95 | 0.96 |
| Gemini-2.5-pro | 400 | 0.94 | 0.01 | 0.00 | 0.93 | 0.95 |
| Gemini-2.5-pro | 790 | 0.92 | 0.02 | 0.01 | 0.91 | 0.93 |
| GPT-5 mini (with thinking) | 1 | 0.98 | 0.00 | 0.00 | 0.98 | 0.98 |
| GPT-5 mini (with thinking) | 10 | 0.98 | 0.00 | 0.00 | 0.97 | 0.98 |
| GPT-5 mini (with thinking) | 25 | 0.97 | 0.00 | 0.00 | 0.96 | 0.97 |
| GPT-5 mini (with thinking) | 50 | 0.96 | 0.00 | 0.00 | 0.96 | 0.97 |
| GPT-5 mini (with thinking) | 100 | 0.96 | 0.01 | 0.00 | 0.96 | 0.96 |
| GPT-5 mini (with thinking) | 150 | 0.97 | 0.01 | 0.00 | 0.96 | 0.97 |
| GPT-5 mini (with thinking) | 200 | 0.96 | 0.01 | 0.00 | 0.95 | 0.96 |
| GPT-5 mini (with thinking) | 400 | 0.87 | 0.12 | 0.04 | 0.78 | 0.96 |
| GPT-5 (with thinking) | 1 | 0.98 | 0.00 | 0.00 | 0.98 | 0.98 |
| GPT-5 (with thinking) | 10 | 0.98 | 0.00 | 0.00 | 0.98 | 0.99 |
| GPT-5 (with thinking) | 25 | 0.98 | 0.00 | 0.00 | 0.98 | 0.98 |
| GPT-5 (with thinking) | 50 | 0.98 | 0.00 | 0.00 | 0.98 | 0.98 |
| GPT-5 (with thinking) | 100 | 0.98 | 0.00 | 0.00 | 0.98 | 0.98 |
| GPT-5 (with thinking) | 150 | 0.98 | 0.00 | 0.00 | 0.98 | 0.98 |
| GPT-5 (with thinking) | 200 | 0.98 | 0.00 | 0.00 | 0.98 | 0.99 |

**Geometric Mean √(Sensitivity × Specificity)**

| AI Model | Batch Size | Mean | Std Dev | Std Error | Lower 95% CI | Upper 95% CI |
| --- | --- | --- | --- | --- | --- | --- |
| Gemini-2.5-flash | 1 | 0.91 | 0.00 | 0.00 | 0.91 | 0.91 |
| Gemini-2.5-flash | 10 | 0.95 | 0.01 | 0.00 | 0.94 | 0.95 |
| Gemini-2.5-flash | 25 | 0.94 | 0.01 | 0.00 | 0.94 | 0.95 |
| Gemini-2.5-flash | 50 | 0.95 | 0.01 | 0.00 | 0.94 | 0.95 |
| Gemini-2.5-flash | 100 | 0.94 | 0.02 | 0.01 | 0.92 | 0.96 |
| Gemini-2.5-flash | 150 | 0.95 | 0.02 | 0.01 | 0.94 | 0.96 |
| Gemini-2.5-flash | 200 | 0.95 | 0.01 | 0.00 | 0.94 | 0.96 |
| Gemini-2.5-flash | 400 | 0.96 | 0.01 | 0.00 | 0.95 | 0.96 |
| Gemini-2.5-pro | 1 | 0.98 | 0.00 | 0.00 | 0.98 | 0.98 |
| Gemini-2.5-pro | 10 | 0.97 | 0.00 | 0.00 | 0.97 | 0.98 |
| Gemini-2.5-pro | 25 | 0.98 | 0.00 | 0.00 | 0.97 | 0.98 |
| Gemini-2.5-pro | 50 | 0.97 | 0.00 | 0.00 | 0.97 | 0.98 |
| Gemini-2.5-pro | 100 | 0.98 | 0.00 | 0.00 | 0.97 | 0.98 |
| Gemini-2.5-pro | 150 | 0.98 | 0.00 | 0.00 | 0.98 | 0.98 |
| Gemini-2.5-pro | 200 | 0.97 | 0.01 | 0.00 | 0.97 | 0.98 |
| Gemini-2.5-pro | 400 | 0.96 | 0.01 | 0.00 | 0.96 | 0.97 |
| Gemini-2.5-pro | 790 | 0.94 | 0.01 | 0.00 | 0.94 | 0.95 |
| GPT-5 mini (with thinking) | 1 | 0.98 | 0.01 | 0.00 | 0.97 | 0.98 |
| GPT-5 mini (with thinking) | 10 | 0.98 | 0.00 | 0.00 | 0.98 | 0.99 |
| GPT-5 mini (with thinking) | 25 | 0.98 | 0.00 | 0.00 | 0.98 | 0.98 |
| GPT-5 mini (with thinking) | 50 | 0.98 | 0.00 | 0.00 | 0.97 | 0.98 |
| GPT-5 mini (with thinking) | 100 | 0.97 | 0.00 | 0.00 | 0.97 | 0.97 |
| GPT-5 mini (with thinking) | 150 | 0.97 | 0.01 | 0.00 | 0.96 | 0.98 |
| GPT-5 mini (with thinking) | 200 | 0.92 | 0.05 | 0.02 | 0.88 | 0.96 |
| GPT-5 mini (with thinking) | 400 | 0.65 | 0.04 | 0.01 | 0.62 | 0.68 |
| GPT-5 (with thinking) | 1 | 0.97 | 0.01 | 0.00 | 0.96 | 0.97 |
| GPT-5 (with thinking) | 10 | 0.98 | 0.01 | 0.00 | 0.98 | 0.99 |
| GPT-5 (with thinking) | 25 | 0.98 | 0.01 | 0.00 | 0.98 | 0.99 |
| GPT-5 (with thinking) | 50 | 0.98 | 0.00 | 0.00 | 0.98 | 0.99 |
| GPT-5 (with thinking) | 100 | 0.98 | 0.01 | 0.00 | 0.97 | 0.98 |
| GPT-5 (with thinking) | 150 | 0.98 | 0.01 | 0.00 | 0.97 | 0.98 |

**Gemini 2.5 Flash Detailed Results**


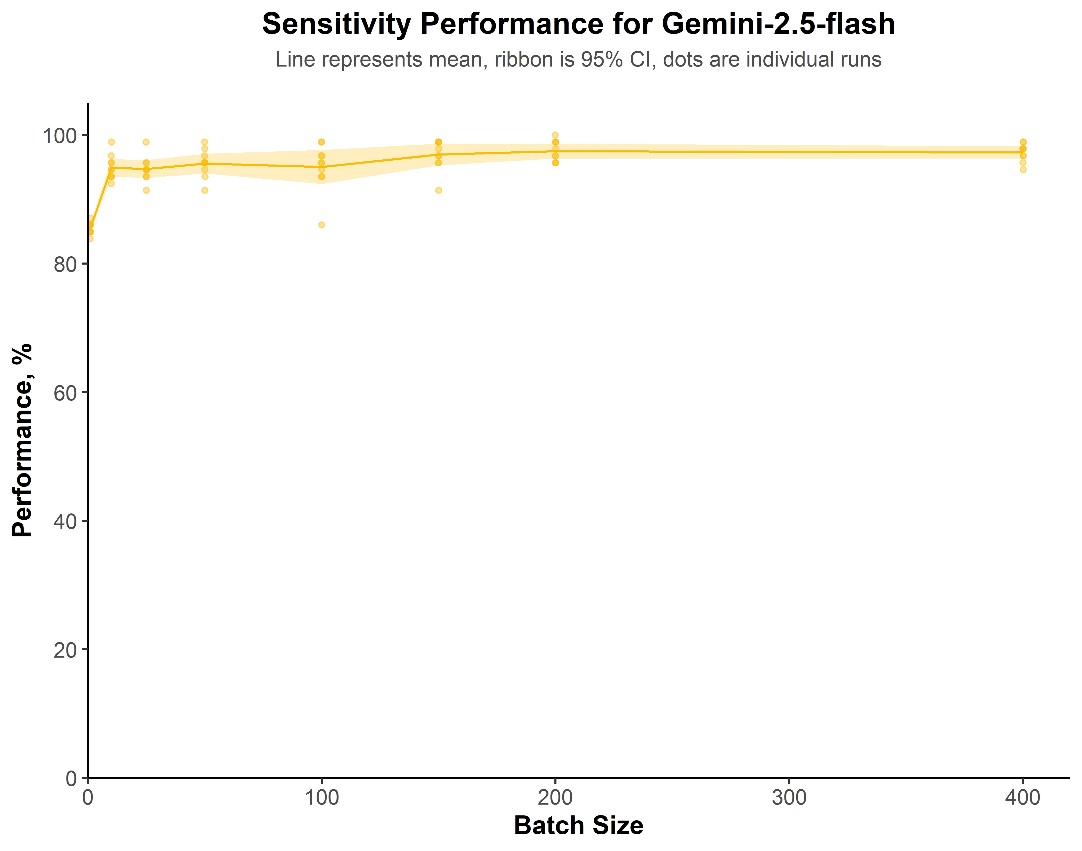


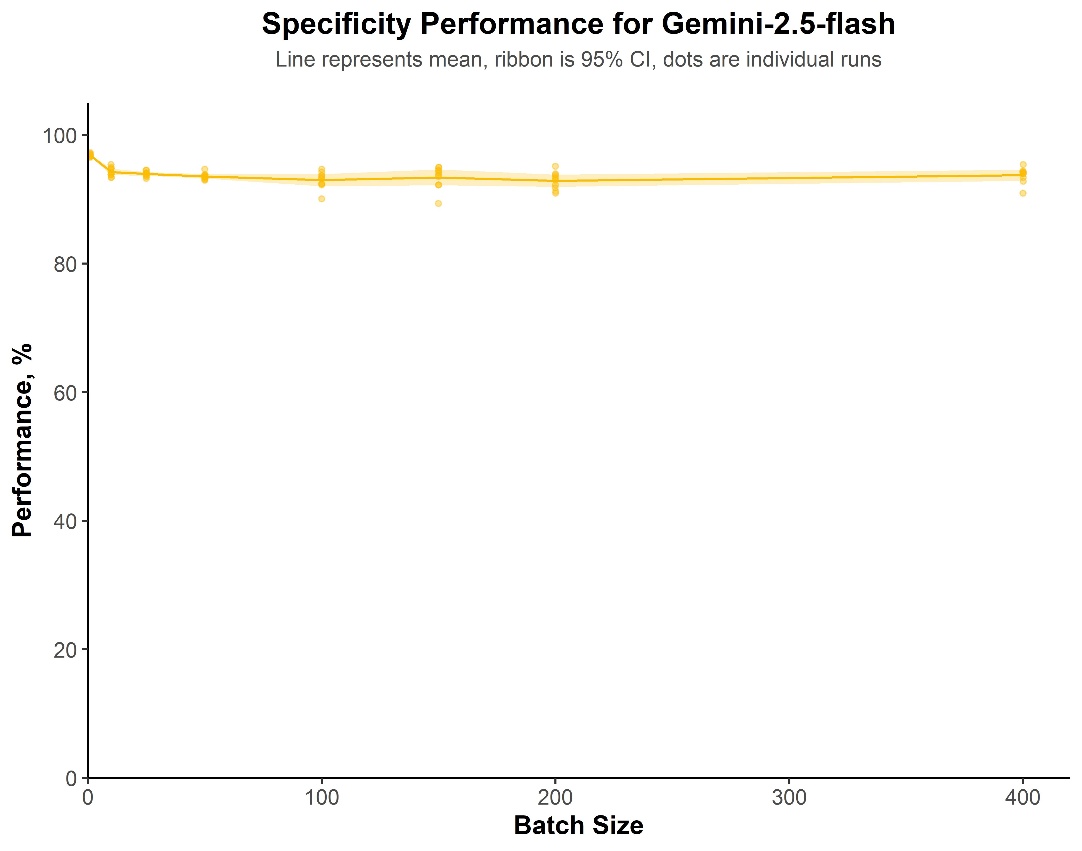


**2.5 Pro Detailed Results**

**
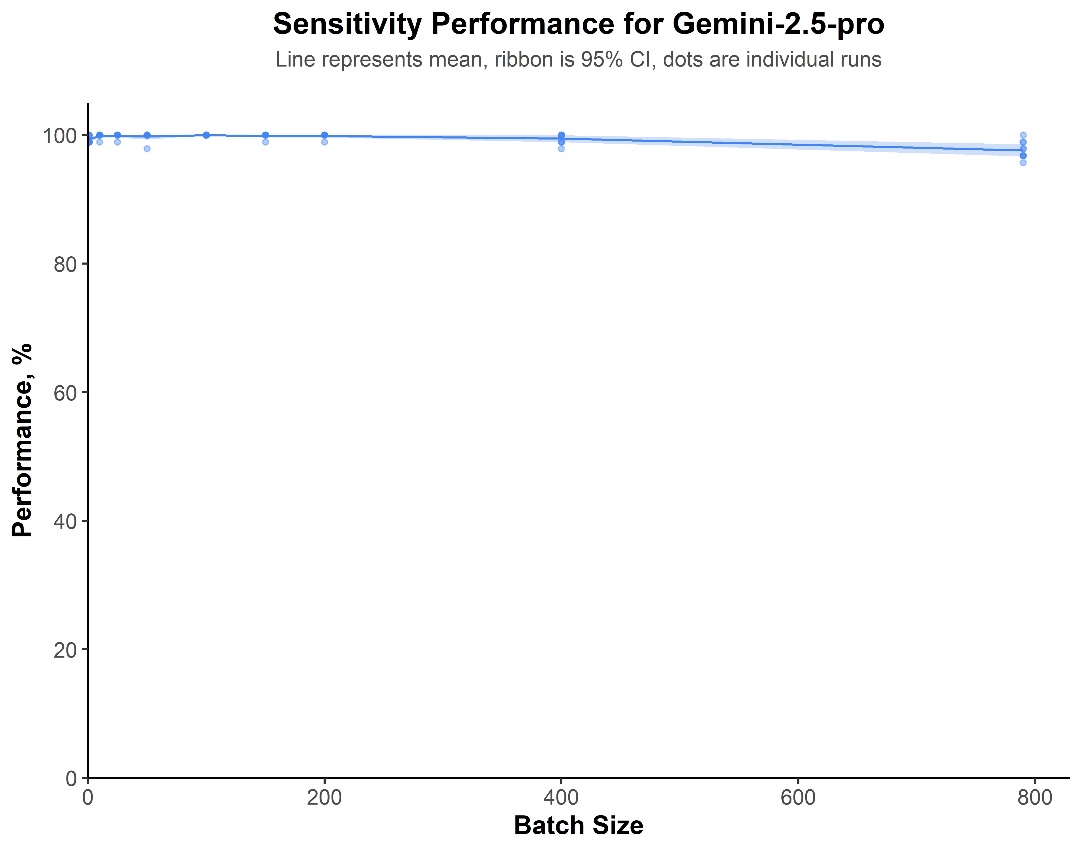
**

**
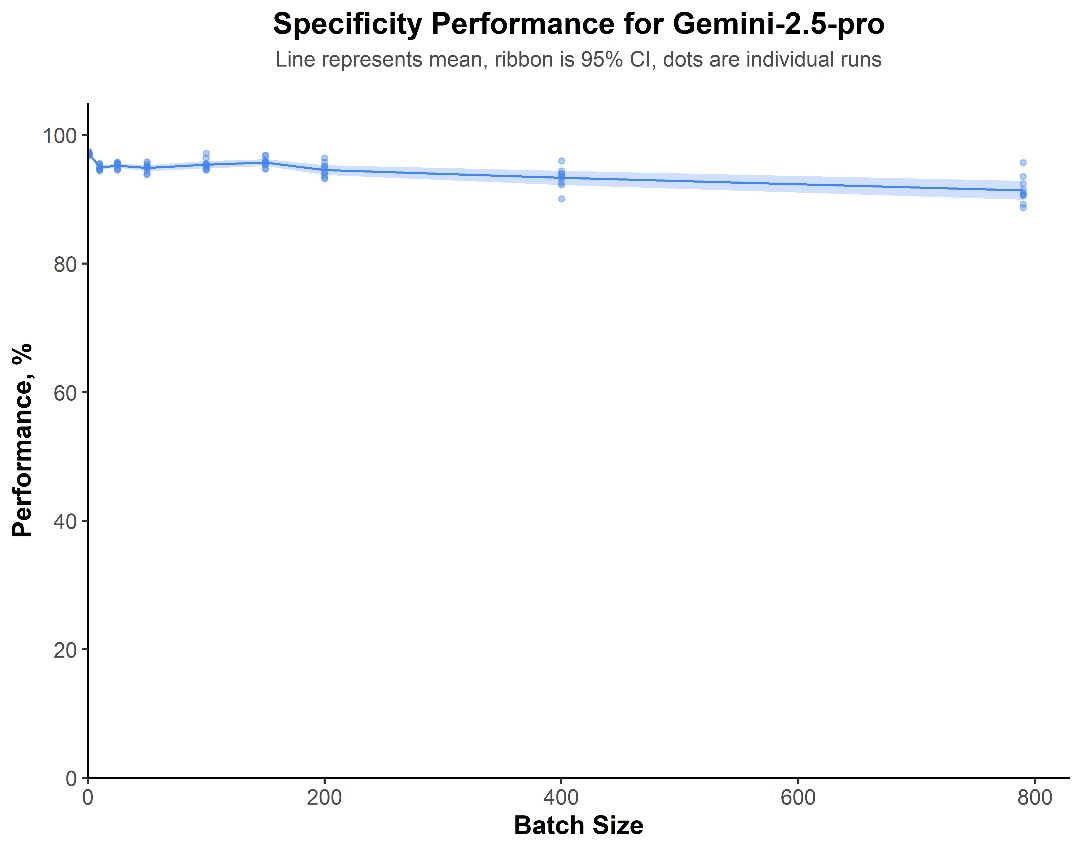
**

**GPT-5 (with thinking) Detailed Results**

**
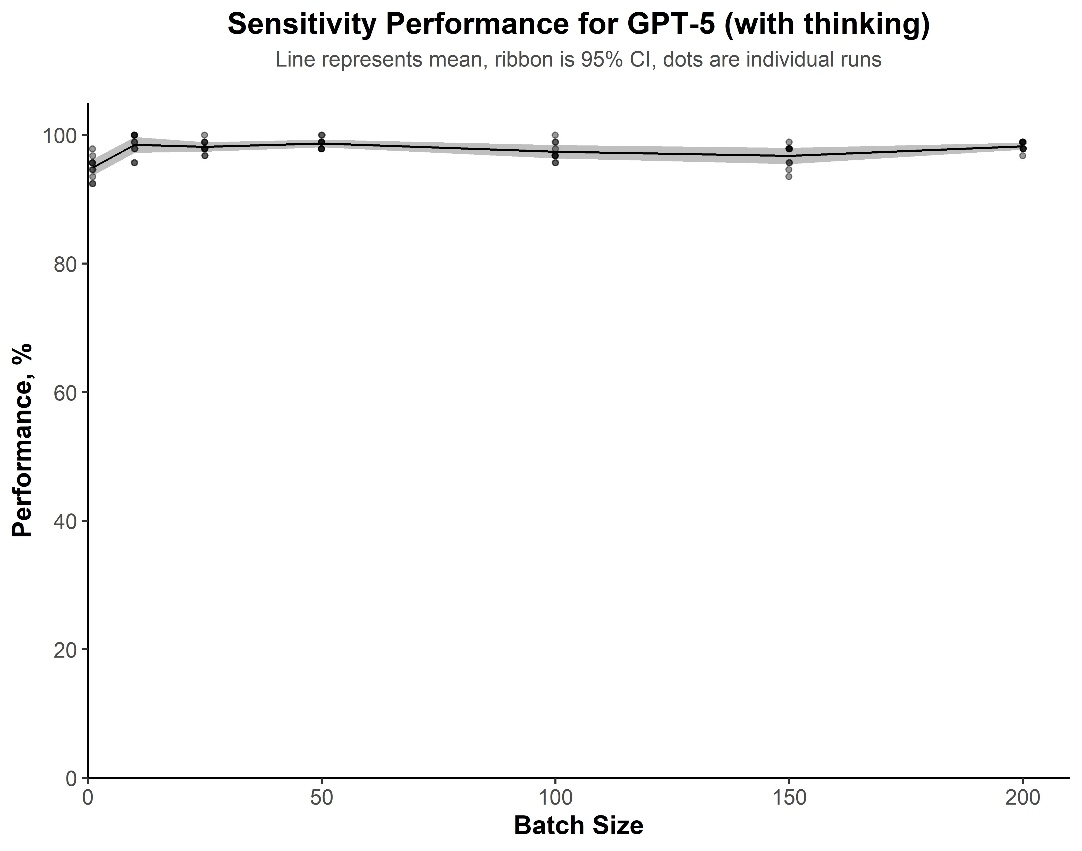
**

**
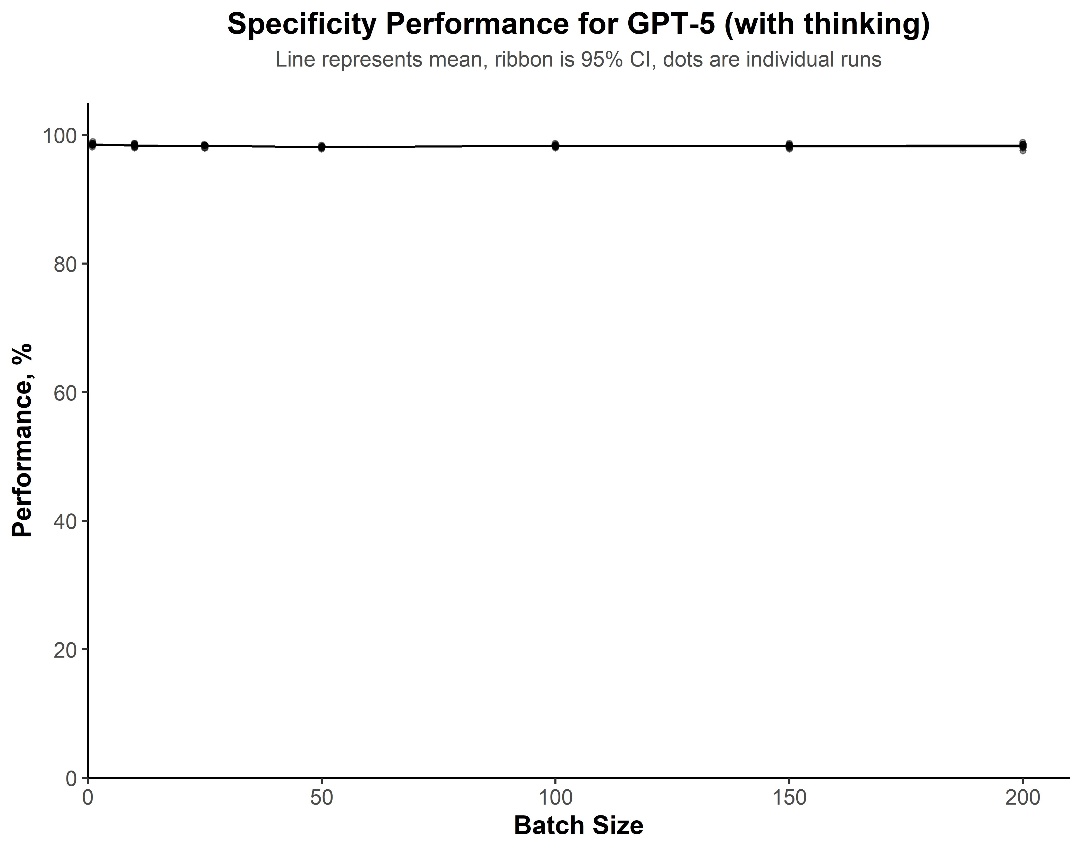
**

**GPT-5 mini (with thinking) Detailed Results**

**
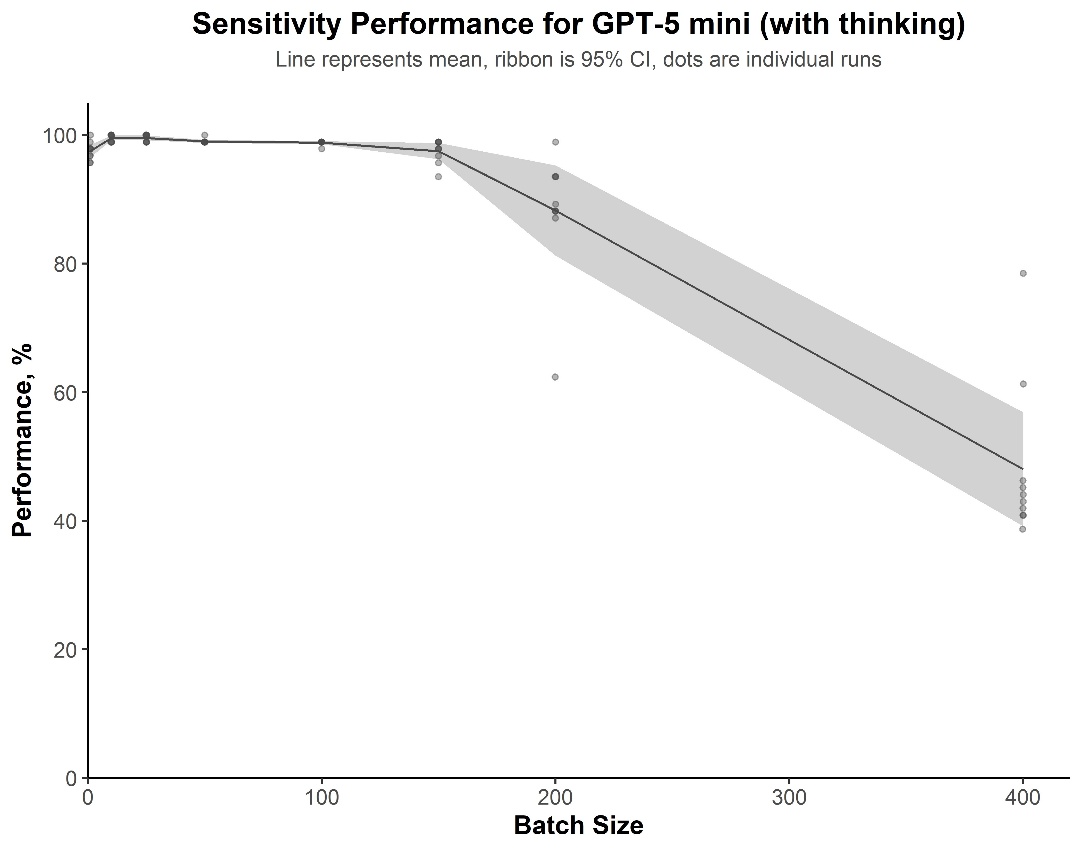
**

**
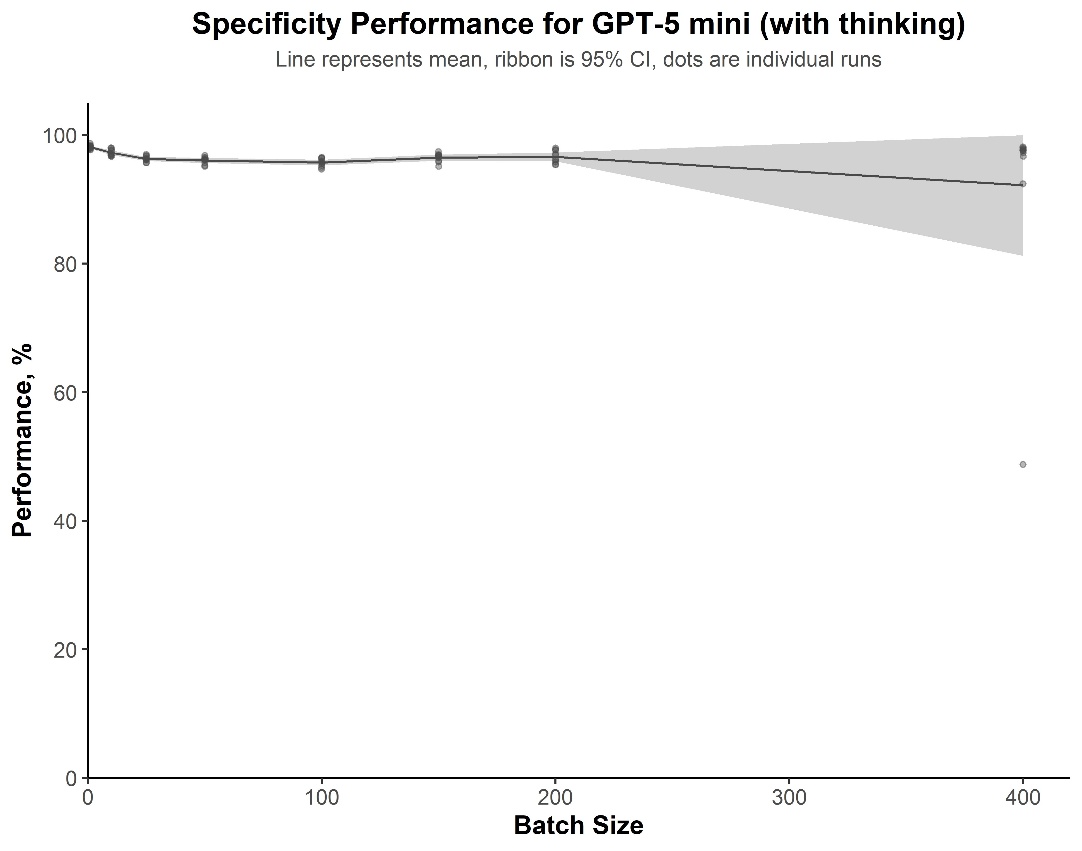
**

**Sensitivity and specificity for all tested models, including legacy models from OpenAI (o3 and GPT-4.1)**

**
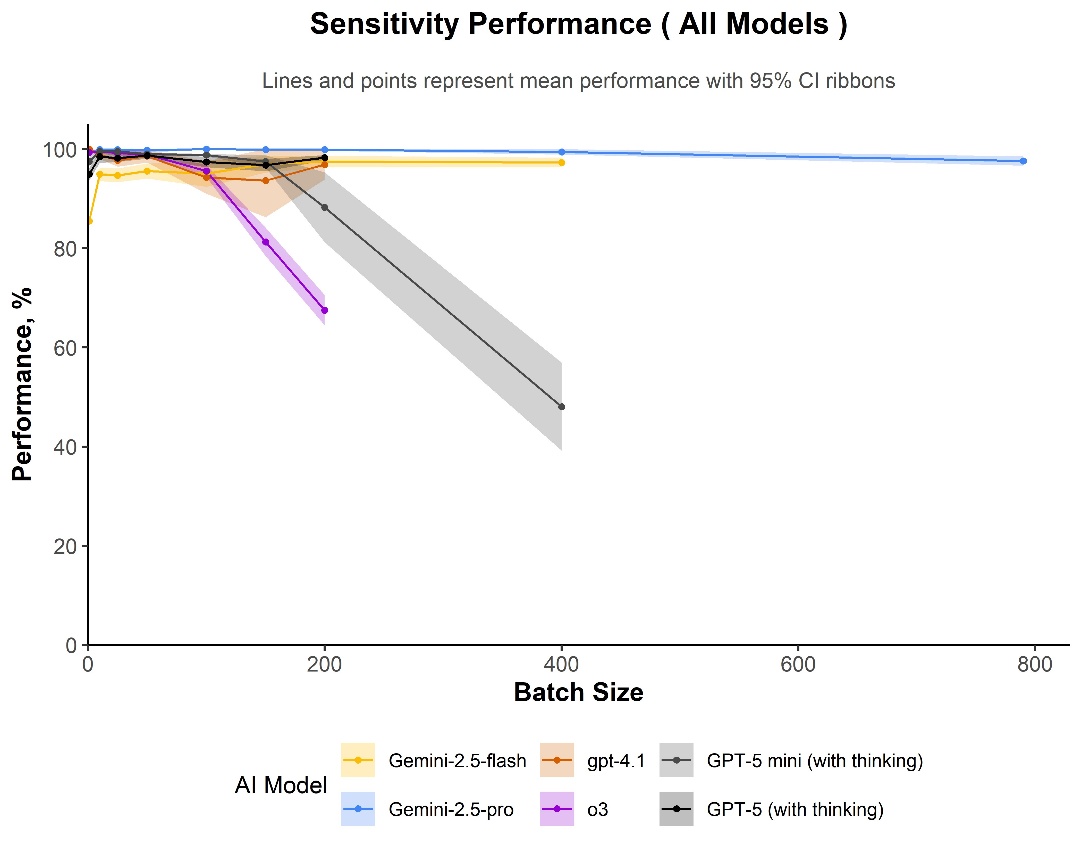
**

**
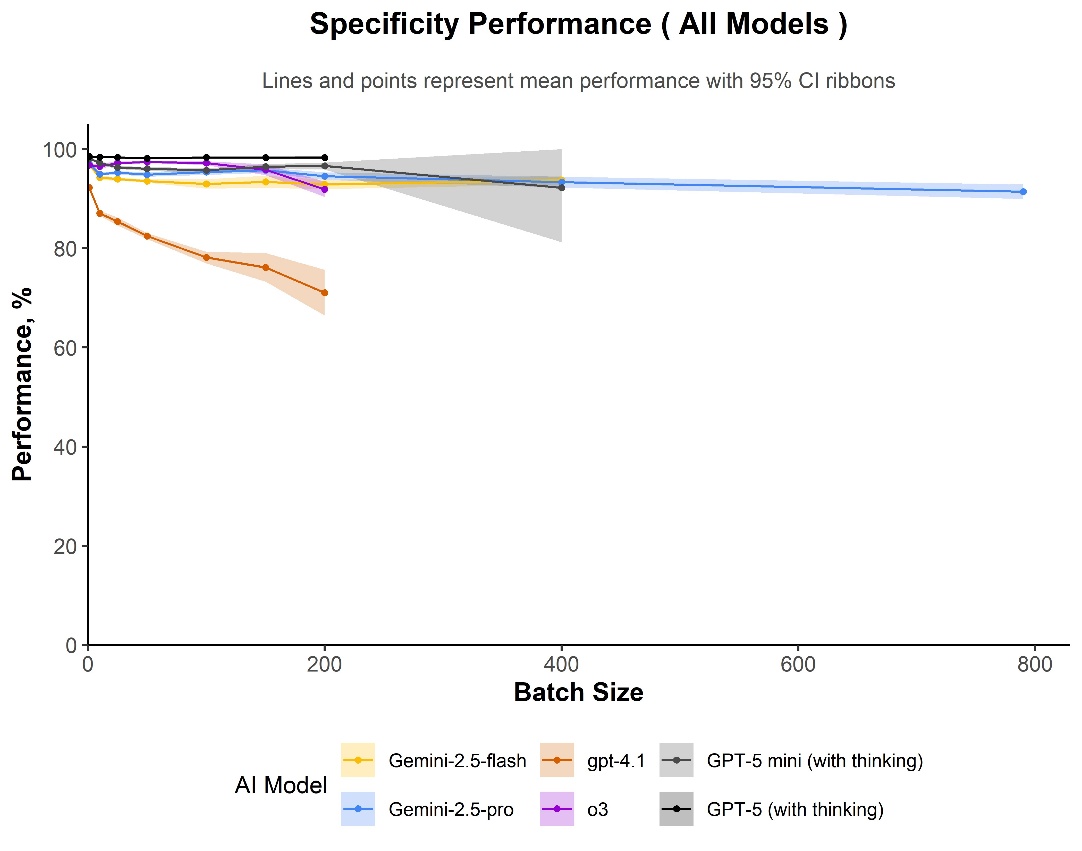
**

**Adjudication of citations initially classified as 'Include' by the Cochrane authors**

| Study | AI ‘Exclude’ reason | Final adjudication decision |
| --- | --- | --- |
| Intracoronary stem-cell injection after myocardial infarction: microcirculation sub-study [20] | “No eligible comparator (both arms received BMMC) and outcomes limited to cell distribution rather than clinical or LVEF.” | While the publication is a sub-study of an eligible RCT, it reports on outcomes (cell retention and safety) outside the review's eligibility criteria. The record was therefore re-classified as 'Exclude'. |
| Intracoronary infusion of autologous mononuclear bone marrow cells in patients with acute myocardial infarction treated with primary PCI: Pilot study of the multicenter HEBE trial [21] | “Not a randomized controlled trial; single-arm pilot without a control group.” | The publication describes a non-randomized pilot study for the HEBE trial. As it failed to meet the randomization criteria, it was re-classified as 'Exclude'. |
| Impaired therapeutic efficacy of bone marrow cells from post-myocardial infarction patients in the TIME and LateTIME clinical trials [22] | “Not an RCT in AMI patients; this is a preclinical mouse study using human post-MI bone marrow cells.” | Although the study relates to the eligible TIME and LateTIME human trials, this publication describes a pre-clinical mouse model. It was re-classified as 'Exclude' for using a non-human population. |
| Bone marrow characteristics associated with changes in infarct size after STEMI: a biorepository evaluation from the CCTRN TIME trial [23] | “Secondary biorepository analysis from the TIME RCT focusing on infarct size only (non-eligible outcome) without randomized intervention comparison.” | The study was a non-randomized prospective cohort, and its reported outcomes did not meet the eligibility criteria. It was subsequently re-classified as 'Exclude'. |
| REPAIR-AMI: stem cells for acute myocardial infarction [24] | “Commentary/review of REPAIR-AMI rather than a primary RCT report; no original patient data.” | The publication is a review article, not a report of original research. As the protocol requires primary studies, it was re-classified as 'Exclude'. |

# Systematic literature search documentation

**Database:** Cinahl **Database provider:** Ebsco **Date:** 2025-06-07

Table shows search strategy with search terms and number of references.

| **Söknr** | **Termtyp** | **Search terms** | **Antal träffar** |
| --- | --- | --- | --- |
|  | DE | (MH "Cell Transplantation+") |  |
|  | DE | (MH "Stem Cells+") |  |
|  | FT/TI, AB | TI ( (stem or haematopoietic or hematopoietic or haematopoetic or hematopoetic or hemopoietic or haemopoietic or progenitor or precursor or bone marrow or mononuclear or adipose tissue or mesenchymal or stromal or autologous or allogeneic or allogenic or ALDH* or C-KIT*)N2 cell* ) OR AB ( (stem or haematopoietic or hematopoietic or haematopoetic or hematopoetic or hemopoietic or haemopoietic or progenitor or precursor or bone marrow or mononuclear or adipose tissue or mesenchymal or stromal or autologous or allogeneic or allogenic or ALDH* or C-KIT*) N2 cell) |  |
|  | FT/TX | TX ( (autologous N3 transplant*) or cell* therap* ) |  |
|  | FT/TI, AB | TI ( (cell* or myoblast*) N3 (autologous or transplant* or autotransplant* or allotransplant* or graI* or implant*) ) OR AB ( (cell* or myoblast*) N3 (autologous or transplant* or autotransplant* or allotransplant* or graI* or implant*) ) |  |
|  |  | 1 OR 2 OR 3 OR 4 OR 5 |  |
|  | DE | (MH "Heart Diseases+") |  |
|  | FT/TI, AB | TI ( (myocardial or myocardium or subendocardial or transmural or cardiac or cardial or coronary or heart or acute) N3 (infarct* or postinfarct* or hypoxi* or anoxi*) ) OR AB ( (myocardial or myocardium or subendocardial or transmural or cardiac or cardial or coronary or heart or acute) N3 (infarct* or postinfarct* or hypoxi* or anoxi*) ) |  |
|  | FT/TI, AB | TI ( ("heart disease*" or "coronary disease*" or IHD or CIHD or DCM or IDCM) ) AND AB ( ("heart disease*" or "coronary disease*" or IHD  or CIHD or DCM or IDCM) ) |  |
|  | FT/TI, AB | TI ( ((myocardial N3 dysfunction) OR angina OR stenocardia) ) OR AB ( ((myocardial N3 dysfunction) OR angina OR stenocardia) ) |  |
|  | FT/TI, AB | TI ( ((ischemi* or ischaemi* or nonischemi* or nonischaemi*) N5 (myocardium or myocardial or heart or coronary or cardiac or cardial or subendocardial or cardiomyopath*)) ) OR AB ( ((ischemi* or ischaemi* or nonischemi* or nonischaemi*) N5 (myocardium or myocardial or heart or coronary or cardiac or cardial or subendocardial or cardiomyopath*)) ) |  |
|  | FT/TI, AB | TI(((arter* occlusion* or arter* disease* or arterioscleros* or atheroscleros*) N2 coronary))OR AB(((arter* occlusion* or arter* disease*or arterioscleros* or atheroscleros*) N2 coronary) ) |  |
|  | FT/TI, AB | TI ( ((myocardial or myocardium or subendocardial or transmural or cardiac or cardial or coronary or heart) N2 (failure* or decompensation or insuNicien*)) ) OR AB ( ((myocardial or myocardium or subendocardial or transmural or cardiac or cardial or coronary or heart) N2 (failure* or decompensation or insuNicien*)) ) |  |
|  | FT/TI, AB | TI ( (end stage or endstage or dilated or idiopathic or congestive) N2 cardiomyopath* ) OR AB ( (end stage or endstage or dilated or idiopathic or congestive) N2 cardiomyopath* ) |  |
|  | FT/TI, AB | TI ( (heart or cardiac or cardial or myocardium or myocardial) N3 (repair* or reparation or improv* or regenerat*) ) OR AB ( (heart or cardiac or cardial or myocardium or myocardial) N3 (repair* or reparation or improv* or regenerat*) ) |  |
|  | FT/TI, AB | TI (heart attack* or coronary attack* or acute coronary syndrome* or AMI) OR AB (heart attack* or coronary attack* or acute coronary syndrome* or AMI) |  |
|  |  | S7 OR S8 OR S9 OR S10 OR S11 OR S12 OR S13 OR S14 OR S15 OR S16 |  |
|  |  | S6 AND S17 |  |
|  | FT/TI, AB | TI ( cellular cardiomyoplast* or ((cardiomyocyte* or cardiac cell*) N6 transplant*) or ((intramyocardial* or intracoronary or transendocardial* or transcoronary) N6 (transplant* or stem or bone marrow or marrow cell* or BMC* or stromal or mesenchymal or progenitor cell* or precursor cell*)) ) OR AB ( cellular cardiomyoplast* or ((cardiomyocyte* or cardiac cell*) N6 transplant*) or ((intramyocardial* or intracoronary or transendocardial* or transcoronary) N6 (transplant* or stem or bone marrow or marrow cell* or BMC* or stromal or mesenchymal or progenitor cell* or precursor cell*)) |  |
|  |  | S18 OR S19 |  |
|  | DE | (MH CLINICAL TRIALS+) |  |
|  | DE | PT Clinical Trial |  |
|  | FT/TI, AB | TI ((controlled trial*) or (clinical trial*)) OR AB ((controlled trial*) or (clinical trial*)) |  |
|  | FT/TI, AB | TI ((singl* blind*) OR (doubl* blind*) OR (trebl* blind*) OR (tripl* blind*) OR (singl* mask*) OR (doubl* mask*) OR (tripl* mask*)) OR AB ((singl* blind*) OR (doubl* blind*) OR (trebl* blind*) OR (tripl* blind*) OR (singl* mask*) OR (doubl* mask*) OR (tripl* mask*)) |  |
|  | FT/TI, AB | TI randomi* OR AB randomi* |  |
|  | DE | MH RANDOM ASSIGNMENT |  |
|  | FT/TI, AB | TI ((phase three) or (phase III) or (phase three)) or AB ((phase three) or (phase III) or (phase three)) |  |
|  | FT/TI, AB | ( TI (random* N2 (assign* or allocat*)) ) OR ( AB (random* N2 (assign* or allocat*)) ) |  |
|  | DE | MH PLACEBOS |  |
|  | FT/TI, AB | TI placebo* OR AB placebo* |  |
|  | DE | MH QUANTITATIVE STUDIES |  |
|  |  | S21 or S22 or S23 or S24 or S25 or S26 or S27 or S28 or S29 or S30 or S31 |  |
|  |  | S20 and S32 Published Date: 20201231-20220202 | **847** |

DE = Descriptor – Established subject heading. FT/TI, AB = Free-text search in the title and abstract fields. MH = Exact Subject Heading –Specific term describing the content of the article.MM = Exact Major Subject Heading – Describes the mani focus of the article. SU = Subject terms – Searches keywords, subject headings, and geographic terms. + = The term is searched including the more specific subordinate terms beneath it. ZC = Methodology Index. ” ” = Quotation marks; searches for an exact phrase. * = Truncation – Shortening a word to capture different inflections and word endings.
